# Peer Toy Play as a Gateway to Children’s Gender Flexibility: The Effect of (Counter)Stereotypic Portrayals of Peers in Children’s Magazines

**DOI:** 10.1007/s11199-017-0883-3

**Published:** 2018-01-23

**Authors:** Lauren Spinner, Lindsey Cameron, Rachel Calogero

**Affiliations:** 1 School of Psychology, University of Kent, Canterbury, Kent CT2 7NP UK; 2 Department of Psychology, University of Western Ontario, London, ON Canada

**Keywords:** Early childhood development, Stereotyped behavior, Gender role attitudes, Gender flexibility, Toy play, Media exposure, Children’s print magazines, Social acceptance, Bullying

## Abstract

Extensive evidence has documented the gender stereotypic content of children’s media, and media is recognized as an important socializing agent for young children. Yet, the precise impact of children’s media on the endorsement of gender-typed attitudes and behaviors has received less scholarly attention. We investigated the impact of stereotypic and counter-stereotypic peers pictured in children’s magazines on children’s gender flexibility around toy play and preferences, playmate choice, and social exclusion behavior (*n* = 82, age 4–7 years-old). British children were randomly assigned to view a picture of a peer-age boy and girl in a magazine playing with either a gender stereotypic or counter-stereotypic toy. In the stereotypic condition, the pictured girl was shown with a toy pony and the pictured boy was shown with a toy car; these toys were reversed in the counter-stereotypic condition. Results revealed significantly greater gender flexibility around toy play and playmate choices among children in the counter-stereotypic condition compared to the stereotypic condition, and boys in the stereotypic condition were more accepting of gender-based exclusion than were girls. However, there was no difference in children’s own toy preferences between the stereotypic and counter-stereotypic condition, with children preferring more gender-typed toys overall. Implications of the findings for media, education, and parenting practices are discussed, and the potential for counter-stereotypic media portrayals of toy play to shape the gender socialization of young children is explored.

Gender-normative attitudes and behaviors, and their accompanying stereotypes, dominate children’s media and popular culture (Blakemore and Centers 2005; Leaper et al. 2002; Murnen et al. 2016; Thompson and Zerbinos 1995). Portrayals of boys tend to emphasize masculine gender roles and stereotypically masculine play and toys, whereas portrayals of girls tend to emphasize feminine gender roles and stereotypically feminine play and toys (Cherney and London 2006; Kahlenberg and Hein 2010). These gendered messages are communicated through various forms of children’s media, including television programming and advertisements (Bakir 2013; Bakir and Palan 2013; Merskin 2002), books (Foster 2016; Skinner 2013), video games (Miller and Summers 2007; Sheldon 2004), and print magazines (Spinner et al. 2016).

Exposure to gender-stereotyped models in children’s media has implications for children’s social and gender-specific development (Coyne et al. 2014; Signorielli 2001). One important domain that has been understudied is the impact of peers on children’s gender flexibility in their preferences for toys and playmates. In the present study, we build on previous investigations of the impact of gendered media on children by testing the effect of exposure to gender-typed toy play by peers pictured in children’s print magazines on gender flexibility in toy and playmate preferences in young children. In particular, we examined the extent to which various indicators of children’s gender flexibility, including gender-based social exclusion, may be undermined and/or bolstered by peers’ (counter)stereotypic displays of toy play through this medium.

## Gender Flexible Attitudes and Behavior

*Gender flexibility* refers to an open-minded attitude around gender roles. Ruble and Martin (1998, p. 947) defined it as “the willingness to apply an attribute to both sexes, rather than just to one or the other, or the recognition of the relativity of stereotypes (e.g., that norms could be different in another culture).” Previous research has shown that gender flexibility is acquired once gender-related knowledge has been established (Huston 1983). So although children’s gender stereotype knowledge rapidly increases between the ages of 3 and 6 years-old (Aubry et al. 1999), their acceptance of these stereotypes as “correct” or “fixed” begins to decline, with gender flexibility peaking and then plateauing at around 7 years of age, following a period of gender stereotype rigidity between the ages of 5 and 6 years (Miller et al. 2006; Huston 1983; Signorella et al. 1993; Trautner 1992).

This trajectory of gender flexibility has been demonstrated by Halim et al. (2014) in their research on gender appearance rigidity among children aged 3–7 years of age. They found that younger girls were more motivated to dress in gender-typed ways than older girls were, and understanding of gender stability (i.e. knowledge that gender remains stable over time) predicted appearance rigidity in both boys and girls. There is also evidence that children’s gender-typed play increases in rigidity between the ages of 3 and 4 years, remaining stable until age 5 (Halim et al. 2013). Similarly, when examining the role of gender development in Halloween costume choices among infants and preschoolers, Dinella (2017) found strong gendered trends in these costumes, with princess costumes being most popular for girls and superhero costumes for boys and with age being positively related to the gender-typing of children’s costumes in this young sample. Together, these findings reflect a strengthening in gender-typed behavior among pre-school children, as well as the emergence of gender flexibility among older children as they approach 7 years of age, as predicted by cognitive developmental theories of gender development.

Flexibility around gender can be expressed in a multitude of ways and directed toward oneself and/or others, with children tending to show more tolerance toward others’ gender-flexible behavior, but less so toward their own gender-flexible behavior (Katz and Ksansnak 1994). Two specific contexts within which children might be able to express gender-flexible behavior include their toy preferences and playmate preferences. Preferences for gender-typed toys and same-gender playmates begin to emerge around 2 years of age (Caldera et al. 1989; Maccoby and Jacklin 1987; Serbin et al. 1994; Wood et al. 2002). The entrenchment of gender stereotypes and prejudice at such an early and formative stage of development has implications for children’s identities, aspirations, and achievements (Cimpian et al. 2012) as well as the perpetration of gender-related bullying, peer victimization, and social exclusion (Killen and Stangor 2001).

Moreover, in westernized societies, gender segregation remains a salient feature of many people’s everyday working and social lives, and it contributes to poor gender relationships (Leaper 1994). Gender segregation of peer groups is one of the most salient aspects of children’s social and cognitive development (Geary and Bjorklund 2000; Killen and Stangor 2001; Maccoby 2002). By 6 years of age, children spend significantly more time playing with children of the same gender compared to the other gender (Maccoby and Jacklin 1987), which can increase gender-typed behavior (Martin and Fabes 2001). In order to maintain gender-segregated peer groups and division among playmates, social exclusion may be necessary. Social exclusion can have severe consequences for children, including reduced academic motivation and success, and a negative impact on mental health and well-being (Buhs et al. 2006). Identifying strategies to encourage mixed-gender and counter-stereotypic play is useful because these experiences expose children to a wider variety of play styles and expand opportunities for cognitive and social development (Fabes et al. 2003). It is therefore important to find ways to encourage mixed-gender friendships in children as a means of attenuating gender-typed behavior. We focus on gender flexibility in the present study as one potentially malleable social-cognitive factor that might improve gender relationships for children now as well as the adults they will later become.

## Children’s Print Magazines as a Gender Socializing Agent

There are a number of theoretical accounts for how gender-related attitudes and behaviors develop and why they are relatively inflexible. According to gender schema theory (Bem 1981, 1983), deeply rooted gender polarization in cultural discourse and social institutions promotes the development of gender-based cognitive schemas in children at an early age whereby children acquire a learned readiness to evaluate, organize, and filter information and behavior in terms of what boys and girls should and should not do (Martin and Ruble 2004). From the perspective of cognitive social learning theorists (Bussey and Bandura 1999, 2004), environmental agents provide and reward models of gender-normative behavior for children to observe and imitate, thereby shaping and reinforcing gender-role attitudes and behavior. Cultivation theory argues that the repetition of themes and stereotypes over time in the media, and television programming specifically, leads viewers to cultivate beliefs about the real world that match with the media content (Gerbner 1998). Together, these theoretical accounts converge on the idea that male and female children are transformed into masculine and feminine adults through a variety of gender socialization forces and processes.

Media represent a powerful socializing agent of gender-role norms because they communicate our cultural definitions of gender normativity in a myriad of formats and settings. To date, much of the research on the impact of gender-stereotypic portrayals in media has been conducted in industrialized westernized societies (Collins 2011). Indeed, despite shifts in the gender roles assumed by women and men in recent decades (Rich 2005), as well as the increased professional achievements of women (Hunt 2004), the United Kingdom, for instance, largely remains a “masculine” society (Hofstede 2001). Despite the fact that the gender pay gap is the lowest it has even been in the United Kingdom, women still earn more than 18% less than their male counterparts, and occupations remain highly gender-segregated (Government Equalities Office 2016). The dominant portrayals of women in popular British print magazine advertisements continue to perpetuate gender-stereotyped representations of them (Plakoyiannaki and Zotos 2009). Moreover, the actual and aspirational choices for occupations among young women living in the United Kingdom (*n* = 506; aged 13–18 years-old) continue to reflect deeply entrenched gender roles (Gould 2008), with markedly more female adolescents indicating they want to be models (32%) or actors (29%) compared to engineers (4%) or scientists (14%). This sexist cultural context provides an important site for investigation of the impact of gender-stereotypic portrayals in children’s media and how we might attenuate it.

Children’s magazines represent a print-based medium that remains popular among young children, with approximately 1.8 million children’s magazines being sold in the United Kingdom in 2015 (Statista 2016a) and 45% of 5- to 7-year-olds in the United Kingdom being classified as regular readers of magazines, books, or comics (Statista 2016b). Children’s magazines present gender stereotypes through the images, activities, emotions, colors, advertisements, and narratives featured in the pages. A unique feature of children’s magazines is the use of reader’s pages, which feature photos of actual readers of the magazine and information about them, as opposed to fictional and/or less identifiable characters. Shutts et al. (2010) demonstrated that children prefer objects and activities endorsed by models of the same gender and age as themselves, even though children fail to acknowledge the influence of these social categories on their decisions. We also know that peers are strong enforcers of gender-normative play (Kornienko et al. 2016). We propose that portrayals of age-matched peers who share an interest with readers through the magazine may serve as effective social models for the communication of gender-typed attitudes and behaviors in media aimed at young children, especially regarding gender-typed toy play.

## Toys as Socializing Cultural Products

Children’s toys represent influential cultural products that are strongly gender-stereotyped (Cherney and London 2006), even in societies with an explicit emphasis on gender equality policies, such as Sweden (Nelson 2005). This pattern is unsurprising given the extent to which many popular toys feature gender-stereotyped characteristics in their design (Blakemore and Centers 2005; Murnen et al. 2016) and are explicitly labeled as “boy toys” or “girl toys” in the marketing of these products and within the stores where they are sold (Auster and Mansbach 2012; Kahlenberg and Hein 2010)—consumers would be hard-pressed to miss the “pink aisle” (targeting girls) in any major toy store. Findings from experimental studies indicate that children prefer gender-typed toys in terms of both their function and color (Weisgram et al. 2014; Wong and Hines 2015). For example, Weisgram et al. (2014) found that boys prefer masculine to feminine toys and that girls dislike masculine toy and color combinations more than any other toy type and color combination. Research has also shown that children’s toy preferences are influenced by the *way* in which toys are modeled and *who* is modeling them (Bradbard and Endsley 1983). Children favor novel toys when they are identified with the children’s own gender, and toys modeled by a same-gender child are rated as more attractive (Shell and Eisenberg 1990).

This gender divide in toy preferences merits scientific and practitioner interest because different types of toys facilitate different types of play, and play types have been associated with different developmental trajectories for social and cognitive skills in children. Research with young children has shown that traditional toys for boys (e.g., cars, video games) facilitate the development of visuo-spatial skills and promote a more agentic orientation toward self and others (De Lisi and Wolford 2002; Jirout and Newcombe 2015), whereas traditional toys for girls (e.g., dolls, Disney princesses) facilitate the development of nurturing and empathy skills and promote a more communal and appearance-focused orientation toward self and others (Coyne et al. 2016; Dittmar et al. 2006; Li and Wong 2016). In addition, there is evidence that children’s cultural products, including toys, are becoming more sexualized in gender-divergent ways (Boyd and Murnen 2017; Zurbriggen and Roberts 2013). One study has also linked gender-stereotyped toy play to the career cognitions of 4–7 year-old children (Sherman and Zurbriggen 2014). Specifically, girls who played with Barbie indicated fewer future career options for themselves compared to what they indicated for boys, whereas girls who played with Mrs. Potato Head did not indicate such differences in future career options. Thus, the toys with which children prefer to play matters for their overall development.

## Counter-Stereotypic Models and Gender-Flexibility

Research findings suggest that gender-typed toy preferences and attitudes are malleable and can change in response to exposure to gender counter-stereotypic models (Abad and Pruden 2013; Steyer 2014). Indeed, if stereotypic portrayals and models provide one mode of gender socialization, then counter-stereotypic portrayals and models provide another mode of gender socialization. For example, after a brief exposure to counter-stereotypic portrayals of women in television commercials (vs. stereotypic portrayals), both girls and boys reported less gender-typed views toward women (Pingree 1978). After one exposure to counter-stereotypic (vs. stereotypic) portrayals of female characters in children’s books, both girls and boys demonstrated stronger preferences for gender-neutral toys compared to gender-typed toys (Ashton 1983). However, the strength of the effect of counter-stereotypic models on these preferences and attitudes may be moderated by children’s own gender.

For instance, focusing specifically on highly gender-typed children over a 4-month period, researchers demonstrated a significant shift away from gender-typed toy play after exposure to fictional stories featuring gender-neutral and gender counter-stereotypic toy play, but only for girls (Green et al. 2004). Pike and Jennings (2005) further demonstrated that young participants exposed to 3 min of video footage depicting “real children” engaged in toy play in traditional (all boys) or nontraditional (all girls) settings in television commercials were more likely to categorize toys as appropriate for “both boys and girls” if they have seen the nontraditional commercial, and this effect was stronger for boys than for girls. Additionally, research has shown that boys are more likely to imitate same-gender models than girls are, and boys have been found to be particularly reluctant to imitate female models or male models if they are performing gender-atypical behaviors (Bauer 1993; Perry and Bussey 1979; Slaby and Frey 1975).

Adherence to gender-typed toy play has been found to be particularly strong among boys, because boys are discouraged from play aligned with feminine stereotypes whereas girls are encouraged to play in masculine-typed ways to raise their status (Cahill and Adams 1997). In relation to gender-typed colors, previous research has shown that although boys increasingly avoid pink during the early years of development, there is no evidence to show that girls avoid blue (LoBue and DeLoache 2011). Similarly, although pre-school children have been shown to categorize occupations in line with gender stereotypes (Blakemore 2003; Liben et al. 2002), young children often permit women to occupy masculine-typed occupations, but do not permit men to occupy feminine-typed occupations (Schuette et al. 2012).

Encounters with counter-stereotypic gender-related behavior may also impact gender-related attitudes and behavior beyond toy play preferences. Research has demonstrated that self-perceptions, interests, and pursuits are affected by exposure to gender counter-stereotypic models. For example, 111 3rd and 4th grade boys and girls exposed over a 4-week period to female protagonists in children’s books who displayed gender-atypical behavior increased the number of activities and occupations they identified as gender-appropriate for women to undertake (Scott and Feldman-Summers 1979). Children who were assigned gender-neutral textbooks to practice their reading later judged more activities as appropriate for girls and boys than those who were assigned gender-typed textbooks (Karniol and Gal-Disegni 2009). Nhundu (2007) also found that girls in Zimbabwe who read biographical stories of women in gender atypical careers adjusted their own career aspirations in non-traditional directions. Overall, given the fact that pervasive portrayals of gender stereotypes more broadly serve to channel and limit children’s interests, experiences, and activities over time (Serbin et al. 1994), these research findings underscore the importance of investigating the potential for counter-stereotypic models and representations of gender-related behavior to increase children’s gender flexibility.

Less research has examined the effects of counter-stereotypic gender portrayals on children’s perceptions of other children and their behavior toward them. In one relevant study, using the Playmate and Play Style Preferences Structured Interview (PPPSI) and cartoon depictions of peers, Pasterski et al. (2011) presented children with a social conflict whereby they had to choose between an other-gender playmate who was playing with a same-gender toy (e.g., for boys, a girl playing with vehicles) or a same-gender playmate who was playing with an other-gender toy (e.g., for boys, a boy playing with a tea set). They demonstrated that boys chose playmates based on the play style of the peer rather than the peer’s gender label, whereas girls chose playmates based on play style *and* peer gender label. Thus, play style, rather than gender alone, may underlie gender-segregated play in children. These findings are consistent with research on the cognitive-behavioral similarity model, which proposes that children can overcome preferences for same-gender peers if there are behavioral similarities with an other-gender peer (Martin et al. 2011). For instance, a boy may display a similar preference for playing with a girl who enjoys trucks as he would for playing with a boy. In other words, children who engage in counter-stereotypic play may be integral to normalizing gender desegregation and gender inclusion.

The overall findings from Pasterski et al.’s (2011) study suggest that a perceived shared interest in a play activity may be a critical piece for cultivating gender flexibility and reducing social exclusion because children’s preferences for gender-typed toys and toy play appear earlier in development and before the emergence of gender-segregated group play (Campbell et al. 2002; Serbin et al. 1994). To date, there is limited research on this possibility in young children. Research with older children has indicated that by the age of 9-years-old, children are aware of the potential for exclusion by their peers if they challenge gender-stereotypic group norms by engaging in counter-stereotypic activities, especially if boys try to engage in female-stereotypic activities (Mulvey and Killen 2015). It is less desirable for boys to exhibit feminine behavior or engage in feminine activities than it is for girls to exhibit masculine behavior or engage in masculine activities, and therefore boys are more likely than girls are to be penalized and excluded by peers for breaking from gender norms regarding activity choices (Blakemore 2003; Horn 2008). This pattern suggests that boys may be more likely to make playmate choices based on toy-play, rather than on gender of playmate, whereas girls use gender *and* toy-play information when choosing their playmates.

## The Present Study

The present study integrates and extends previous research on the effects of gender stereotypic versus counter-stereotypic media portrayals of children on a set of gender-flexible attitudes and behavior in young British children. We focused on the impact of portrayals of children engaged in gender-stereotypic or counter-stereotypic toy play in print magazines, depicted in the form of actual children playing with their toys and who were fellow readers of the magazine, that is, in a format made to resemble the content of a Reader’s Page that is often found in children’s magazines. The portrayals of the children included an age-matched male and female child to bolster the validity and potential impact of the peer (Bartini 2006). The children were depicted as playing with a toy deemed appropriate for their own gender (stereotypic toy play) or a toy deemed appropriate for the other gender (counter-stereotypic toy play). This design allowed us to randomly assign children to view (a) magazine content that pictured a boy and girl engaged in stereotypic toy play or (b) magazine content that pictured a boy and girl engaged in counter-stereotypic toy play.

We also used a variety of markers of gender flexibility to assess the degree to which the magazine content would differentially shift the gender-related preferences and attitudes of young children. Specifically, we examined whether exposure to counter-stereotypic (vs. stereotypic) peers through this medium would impact preferences for gender-typed toys (see Hypotheses 1a and 1b), attitudes toward gender-typed toy play (see Hypotheses 2a and 2b), playmate preferences (see Hypotheses 3 a–c), and the endorsement of gender-based social exclusion (see Hypotheses 4a and 4b). The focus on playmate preferences and gender-based social exclusion represent particularly understudied outcomes among this developmental age group of 4–7 year-olds, especially in the context of stereotyped media content exposure. We focused on this age range because it is between these ages that children’s gender identity and gender-related knowledge, attitudes, and behaviors develop significantly (Serbin and Sprafkin 1986; Signorella et al. 1993; Zosuls et al. 2009).

For gender-typed toy preferences, we expected children to make gender-typical toy preferences, as evidenced by an interaction between participant gender and toy type, whereby boys would prefer to play with masculine toys over feminine toys and girls would prefer to play with feminine toys over masculine toys (Hypothesis 1a). We also expected condition to moderate children’s gender-typed toy preferences, predicting a three-way interaction among participant gender, condition, and toy type whereby children in the counter-stereotypic condition would prefer other gender toys more than children in the stereotypic condition would, demonstrating greater gender flexibility around toy type (Hypothesis 1b).

For attitudes toward gender-typed toy play, we expected a main effect of participant gender whereby girls would demonstrate more gender flexible attitudes toward toy play than boys (Hypothesis 2a). We also expected a main effect for condition whereby children in the counter-stereotypic condition, compared to children in the stereotypic condition, would be more likely to label toys as being for both boys and girls, demonstrating more gender flexible attitudes around toy play (Hypothesis 2b).

For gender-typed playmate choice, we expected children to demonstrate more gender flexible attitudes around playmate preferences in the counter-stereotypic condition compared with the stereotypic condition. We expected children to be more likely to choose a same-gender than an other-gender playmate in the stereotypic condition, whereas we did not expect to observe this bias in the counter-stereotypic condition (Hypothesis 3a). Also in the counter-stereotypic condition, we expected that boys would be more likely than girls would be to choose an other-gender playmate compared to a same-gender playmate. This is because, compared to girls’, boys’ playmate preference may be more driven by prospective playmates’ toy choice, rather than by their gender, due to more strongly enforced norms for traditional masculine behavior (Hypothesis 3b). We also expected that the reasons children would provide for their playmate choice would be more likely to refer to toy play style than the playmate’s gender in the counter-stereotypic condition, whereas we expected toy play style *and* playmate’s gender to be given as reasons in the stereotypic condition (Hypothesis 3c).

For gender-based social exclusion, we expected children to demonstrate more gender flexible attitudes in the counter-stereotypic versus stereotypic condition. We expected a main effect for condition, whereby children in the counter-stereotypic condition would report less endorsement of gender-based social exclusion than children in the stereotypic condition (Hypothesis 4a), demonstrating more gender flexibility around play groups and less gender-based social exclusion in the counter-stereotypic condition. Finally, we expected an interaction between participant gender and condition whereby in the stereotypic condition boys would report higher gender-based social exclusion scores than girls would, due to stronger disapproval of cross-gender play. In the counter-stereotypic condition, we expected this difference to be attenuated and expected boys and girls to show similar levels of social exclusion (Hypothesis 4b). This is because the counter-stereotypic toy play of the children in this condition makes it less acceptable to exclude them and boys are more likely to be impacted by this behavior.

## Method

### Participants

We recruited 96 British participants who were between the ages of 4–8 years-old. Of this initial sample, 10 participants failed to complete all measures due to time constraints and were not included in the final analysis. In addition, given that only four 8-year-olds completed the study, these data were also not included in the final analysis due to minimal representation of this age group. The final sample for analysis included 82 children (40 boys and 42 girls) aged 4–7 years-old (*M*_age_ = 5.4 years); girls and boys did not significantly differ in age, *t*(80) = .21, *p* = .83. Participants were recruited from an urban primary school in a generally low SES neighborhood. The sample was predominantly White, reflecting the low ethnic diversity in the area. Ethical consent was obtained from the Research Ethics Committee at the University and we complied with British Psychological Society guidelines for research with children. Head teacher, parental, and participant consent were obtained prior to commencement of the study.

### Procedure and Measures

Participants were told that they were going to be shown a magazine page which contained some pictures of children playing with their favorite toys and that they would be asked a few questions about what they thought of the pictures. Participants were reminded that there were no right or wrong answers and that their answers were private. Once verbal consent had been obtained, participants were randomly assigned to the stereotypic or counter-stereotypic condition. In the stereotypic condition, participants viewed a magazine page featuring a male child playing with a car and female child playing with a pony; those in the counter-stereotypic condition viewed a magazine page featuring a male child playing with a pony and a female child playing with a car. Participants viewed the magazine page for 2 min. While the participant viewed the magazine page, the experimenter read aloud the following words from the page: “We love it when you write to us with interesting facts about your life, so this week we have asked our readers to send in photos of them playing with their favorite toys. Check out Sarah and Thomas’ photos below!”

Text in speech bubbles was presented next to the featured male and female children that the experimenter also read aloud. In the stereotypic condition with the female child, the speech bubble read: “Hello! My name is Sarah, and my favorite toy is My Little Pony! I have lots, and play with them every day.” In the stereotypic condition with the male child, the speech bubble read: “Hello! My name is Thomas, and every day I like to play with my cars. They’re my favorite toys!” In the counter-stereotypic condition, the content of the speech bubbles was identical, but the children’s names were switched so that “Sarah” liked to play with cars and “Thomas” liked to play with My Little Pony. These pages are representative of those found in children’s magazines, where children’s photos and letters to the magazine are displayed, or the magazine presents a feature on a reader.

Immediately after viewing the assigned magazine pages, participants completed a series of measures that assessed gender flexible attitudes and behavior. All study materials were presented via Qualtrics on tablet computers. Participants completed the measures individually with an experimenter in a quiet area.

#### Gender-Typed Toy Preferences

To assess gender flexible toy preferences, we presented participants with pictures of eight different toys, including four stereotypically feminine toys (a wand, a pony, a baby doll, and a tea set) and four stereotypically masculine toys (a truck, a jet fighter plane, a tool kit, and a car), based on Blakemore and Centers’s (2005) categorization of toys as “Strongly Feminine Toys” and “Strongly Masculine Toys.” The toys were presented to participants individually and in a randomized order. We coded participants’ responses to the same question for each of the eight toys: “How much do you like this toy?” Participants selected from one of three response options based on a scale depicting schematic faces: “Not at all” (depicted with a frowning face and coded as 1), “A little” (depicted with a slightly smiling face and coded as 2), or “A lot” (depicted with a broadly smiling face and coded as 3). Total scores were calculated separately for the feminine toys (α = .89) and masculine toys (α = .77) by summing the response for the four toys in each category separately. Scores for both types of toys could range from 4 to 12, with higher scores indicating a greater preference for the respective toy type.

#### Gender-Typed Toy Play

To assess gender flexible attitudes around toy play, we coded participants’ responses to the following question for each of the eight toys listed: “Who should play with this toy?” Participants selected from one of three response options, which were also paired with the corresponding gender symbols that appear on restroom signs: “Only Girls” (coded as 0), “Only Boys” (coded as 0), or “Both Girls and Boys” (coded as 1). Participants could respond verbally or by pointing to the symbols of their choice (Weisgram et al. 2014). Total scores were calculated by summing the assigned codes across the eight toys. Scores could range from 0 to 8, with higher scores indicating more gender flexible attitudes toward toy play. It should be noted that none of the participants indicated a counter-stereotypical endorsement (e.g., “only boys should play with dolls”). This means that all responses coded as 0 were stereotypical responses.

#### Gender-Typed Playmate Choice

To assess gender flexible attitudes in playmate choice, participants were presented with pictures of the children they had viewed on the magazine pages (i.e. either the boy and girl engaged in stereotypic or counter-stereotypic toy play) and were asked: “If you had to choose one of the children to play with, which one would you choose, the girl or the boy?” If participants selected a girl playmate this was coded as 0; if they selected a boy playmate this was coded as 1. After selecting a playmate, we coded participants’ responses to the question: “Why would you choose to play with this child?” Responses were coded into categories based on whether they referred to the gender label of the child pictured (coded 1), the type of toy played with by the child pictured (coded 2), or some other feature (coded as 3). It should be noted that none of the participants referred to more than one category in their responses.

#### Gender-Based Social Exclusion

We adapted a measure from Killen and Stangor (2001) to assess gender flexible attitudes around social exclusion. We presented two scenarios to the participants, in a randomized order, and coded their responses. To assess the tendency to exclude the girl from boys’ play, we presented the following scenario:

Imagine that a group of boys are playing with cars. This girl [from the magazine page they viewed] comes over and asks if she can play. Two of the boys say that she cannot play because she is a girl. Is it alright or not alright for the boys to tell the girl that she can’t play?

To assess the tendency to exclude the boy from girls’ play, we presented the following scenario:

Imagine that a group of girls are playing with dolls. This boy [from the magazine page they viewed] comes over and asks if he can play. Two of the girls say that he cannot play because he is a boy. Is it alright or not alright for the girls to tell the boy that he can’t play?

For each scenario, participants selected from one of three response options to indicate the extent to which they believed it was all right to exclude the child from play: “Not alright” (coded 1), “A little bit alright” (coded 2), or “Alright” (coded 3). A total gender-based exclusion score was computed by summing the responses given for the two scenarios. Scores ranged from 2 to 6, with higher scores indicating that gender-based social exclusion was more acceptable.

## Results

### Preliminary Analyses

Power analyses indicated that the statistical tests were sufficiently powered and the sample size was adequate for each planned analysis, with power to find an effect ranging between 74% and 99% across all analyses (Howell 1992). Table 1 presents the overall means and standard deviations for the study variables, as well as the zero-order correlations for the associations among the continuous variables and point-biserial correlations for associations with the dichotomous variable (i.e., gender-typed playmate choice). Correlational analyses were performed separately on the boys’ and girls’ scores to examine initial relationships among the gender flexibility variables by gender group.

**Table 1:** Descriptive statistics and correlations for all study variables as a function of participants’ gender

	Girls	Boys	Correlations					
Variables	*M* (*SD*)	*M* (*SD*)	1	2	3	4	5	6
1. Age	5.38 (.99)	5.43 (.87)	–	−.55**	.09	.56**	.01	−.28
2. Gender-typed feminine toy preference	10.97 (1.39)	6.34 (2.55)	−.25	–	−.12	−.36*	−.03	−.04
3. Gender-typed masculine toy preference	6.34 (2.55)	10.82 (1.39)	−.36*	−.12	–	.35*	−.04	.12
4. Flexibility in gender-typed toy play	3.51 (2.67)	2.74 (2.54)	.41**	.33*	−.03	–	.30	−.27
5. Gender-typed playmate choice	.24 (.44)	.70 (.46)	−.12	−.27	−.16	−.42**	–	−.04
6. Gender-based social exclusion	2.57 (1.23)	3.03 (1.41)	−.39*	−.27	−.04	−.22	.13	–

Values for girls (*n* = 42) are presented above the diagonal; for boys (*n* = 40), below. Point-biserial correlations are reported for the associations with the dichotomous variable of gender-typed playmate choice, where 0 = girl playmate, and 1 = boy playmate. Higher scores indicate greater preference for gender-typed masculine and feminine toys and greater flexibility in gender-typed toy play, whereas higher scores for gender-based social exclusion indicate more exclusion of other-gender playmates, and therefore *less* flexibility in this domain. Higher scores for playmate choice indicate more preference for a boy playmate

*p < .05. **p < .01

A significant positive association was observed between age and flexibility around gender-typed toy play for both boys and girls; as participants’ age increased, they were more likely to believe that both boys and girls should play with both masculine and feminine toys (see Table 1). There was also a significant negative association between age and one’s own gender-typed toy preferences among boys *and* girls; as age increased, boys showed less interest in the masculine toys and girls showed less interest in the feminine toys. Analyses also revealed a significant negative association between age and acceptance of gender-based social exclusion, but only among the boys; as age increased, boys showed less acceptance of gender-based social exclusion across both conditions. No other variables correlated significantly with age. Given these associations with age, we included participants’ age as a covariate in our tests of the main gender flexibility hypotheses.

Several correlations were also observed among the gender flexibility variables for each gender group (see Table 1). Among boys, there was a significant positive relationship between flexibility around gender-typed toy play and feminine toy preference scores, and a significant negative correlation between flexibility around gender-typed toy play and playmate choice; as flexibility around toy play increased, so did the likelihood that boys would choose a female playmate, across both conditions. There were no other significant correlations among the variables for boys. Among girls, analyses revealed a significant negative relationship between flexibility around gender-typed toy play and interest in feminine toys, and a significant positive relationship between flexibility around gender-typed toy play and interest in masculine toys. There were no other significant correlations among the variables for girls. Significant mean gender differences among participants were observed only in the feminine and masculine toy preference scores; these are reported in the following (also see Table 1).

### Primary Analyses

#### Hypotheses for Gender-Typed Toy Preferences

We expected an interaction between participant gender and toy type, whereby boys would prefer to play with masculine toys over feminine toys and girls would prefer to play with feminine toys over masculine toys (Hypothesis 1a). We also expected a three-way interaction between participant gender, condition, and toy type, whereby participants in the counter-stereotypic condition would prefer other-gender toys more than children in the stereotypic condition, demonstrating greater gender flexibility around toy preferences (Hypothesis 1b).

To test this set of hypotheses, we conducted a 2 (Condition: stereotypic vs. counter-stereotypic) × 2 (Participant Gender: girls vs. boys) × 2 (Toy Type: masculine vs. feminine) mixed analysis of covariance (ANCOVA) on ratings of preference for masculine and feminine toys, with participant gender and condition as the between-subjects factors, toy type as the within-subjects factor, and age entered as a covariate. In support of Hypothesis 1a, we observed a significant interaction between participant gender and toy type, *F*(1, 75) = 197.55, *MSE* = 3.33, *p* < .001, ηp^2^ = .73. Pairwise comparisons revealed that girls preferred the feminine toys to the masculine toys (*p* < .001, *d* = 2.21), and boys preferred the masculine toys to the feminine toys (*p* < .001, *d* = 2.27; see Table 2). However, we did not observe support for Hypothesis 1b because the three-way interaction among participant gender, condition, and toy type was not significant, *F* (1, 75) = 1.60, *MSE* = 3.33, *p* = .210, ηp^2^ = .02, suggesting that condition did not affect children’s gender flexibility around toy preferences (see Table 2).

**Table 2:** Gender-typed masculine and feminine toy preference scores as a function of condition and participant gender

		(a) Three-way interaction				(b) Two-way interaction	
		Stereotypic condition		Counter-stereotypic condition		Conditions combined	
Participants’ gender	*n*	Feminine toys *M* (*SD*)	Masculine toys *M* (*SD*)	Feminine toys *M* (*SD*)	Masculine toys *M* (*SD*)	Feminine toys *M* (*SD*)	Masculine toys *M* (*SD*)
Girls	42	11.05 (1.43)	7.68 (1.89)	10.89 (1.37)	6.95 (1.93)	10.97 (1.39)_a_	6.34 (1.92)_b_
Boys	40	6.33 (2.77)	10.44 (1.54)	6.33 (2.42)	11.19 (1.17)	6.34 (2.55)_a_	10.82 (1.39)_b_

The mean differences (i.e., means with different subscripts in a row) between feminine and masculine toys for both female and male participants are significant (*p* < .001.)

#### Hypotheses for Gender-Typed Toy Play

We expected a main effect of participant gender on gender-typed toy play, whereby girls would demonstrate more gender flexible attitudes toward toy play than boys would (Hypothesis 2a). We also expected a main effect for condition, whereby participants in the counter-stereotypic condition would be more likely to label toys as being for both boys and girls compared to participants in the stereotypic condition, demonstrating more gender flexible attitudes around toy play (Hypothesis 2b).

To test this set of hypotheses, we conducted a 2 (Participant Gender) × 2 (Condition) between-subjects ANCOVA on attitudes toward gender-typed toy play, with age entered as a covariate. Refuting Hypothesis 2a, attitudes toward gender-typed toy play did not vary as a function of participants’ gender, *F*(1, 75) = 3.02, *MSE* = 5.03, *p* = .086, ηp^2^ = .04. However, in support of Hypothesis 2b, there was a significant main effect of condition, *F*(1, 75) = 4.29, *MSE* = 5.03, *p* = .042, ηp^2^ = .05, whereby attitudes toward gender-typed toy play were significantly more flexible among participants in the counter-stereotypic condition (*M* = 3.64, *SD* = 2.70) compared to the stereotypic condition (*M* = 2.60, *SD* = 2.45). Participants, regardless of their own gender, were more likely to endorse masculine toys and feminine toys as appropriate for both boys and girls if they had viewed magazine content depicting children playing with counter-stereotypic toys.

#### Hypotheses for Gender-Typed Playmate Choice

We expected that participants would be more likely to choose a same-gender than an other-gender playmate in the stereotypic condition, whereas we did not expect to observe this bias in the counter-stereotypic condition (Hypothesis 3a), thereby demonstrating more gender flexible attitudes around playmate preferences in the counter-stereotypic condition. Also in the counter-stereotypic condition, we expected that boys would be more likely than girls would be to choose an other-gender playmate compared to a same-gender playmate (Hypothesis 3b). We further expected that the reasons participants provide for their playmate choice would more likely refer to toy play style than to the playmate’s gender in the counter-stereotypic condition, whereas we expected toy play style and playmate’s gender to be given as reasons in the stereotypic condition (Hypothesis 3c).

To test this set of hypotheses, we conducted two-way Chi-square tests with Yates correction for continuity to examine the association between participant gender and gender-typed playmate choice for each condition. In support of Hypothesis 3a, in the stereotypic condition, girls were significantly more likely to choose a same-gender playmate (91% vs. 9%) and boys were significantly more likely to choose a same-gender playmate (94% vs. 6%) compared to an other-gender playmate, *χ*^2^(1) = 26.51, *p* < .001, Cramer’s *V* = .85; however this pattern was not observed in the counter-stereotypic condition, where girls (50% vs. 50%) and boys (45% vs. 55%) were equally likely to select an other-gender versus same-gender playmate, *χ*^2^(1) = .00, *p* = 1.00, Cramer’s *V* = .05. However, this finding refutes Hypothesis 3b, because in the counter-stereotypic condition, boys were not more likely than girls to choose an other-gender over a same-gender playmate.

To examine participants’ reasoning behind their playmate preferences, we conducted two one-sample Chi-square tests separately for each condition. As we were primarily interested in whether participants used the child’s play style or their gender as a reason for choosing them as a playmate, reasons which did not fall into one of these two categories (classified as ‘other’) were excluded from analysis (16% of overall reasons in the counter-stereotypic condition; 31% in the stereotypic condition). In support of Hypothesis 3c, participants in the counter-stereotypic condition were significantly more likely to refer to the playmate’s play style (69%) than to the playmate’s gender (31%) when choosing one of the playmates, *χ*^2^(1) = 4.50, *p* = .034, Cramer’s *V* = .38; however this pattern was not observed in the stereotypic condition, where participants were not significantly more likely to refer to the playmate’s play style (66%) over the playmate’s gender (33%), *χ*^2^(2) = 3.00, *p* = .083, Cramer’s *V* = .33.

#### Hypotheses for Gender-Based Social Exclusion

We expected a main effect for condition whereby participants in the counter-stereotypic condition would report less endorsement of gender-based social exclusion than would participants in the stereotypic condition (Hypothesis 4a). Finally, we expected an interaction between participant gender and condition whereby in the stereotypic condition boys would report higher gender-based social exclusion scores than girls would, due to stronger disapproval of cross-gender play. In the counter-stereotypic condition we expected this difference to be attenuated and expected boys and girls to show similar levels of social exclusion (Hypothesis 4b).

To test this set of hypotheses, we conducted a 2 (Condition) × 2 (Participant Gender) between-subjects ANCOVA on gender-based social exclusion scores, with age as a covariate. Counter to our expectations for Hypothesis 4a, the effect of condition was not significant, *F*(1, 77) = .25, *MSE* = 1.52, *p* = .620, ηp^2^ = .00. However, the analysis did reveal a significant interaction between condition and participant gender, *F*(1, 77) = 4.59, *MSE* = 1.52, *p* = .035, ηp^2^ = .06. Supporting Hypothesis 4b, pairwise comparisons revealed significantly higher endorsement of gender-based social exclusion among boys (*M* = 3.27, *SD* = 1.63) compared to girls (*M* = 2.23, *SD* = .64) in the stereotypic condition (*p* = .008, *d* = .92), but not between boys *(M* = 2.82, *SD* = 1.17*)* and girls (*M* = 2.96, *SD* = 1.66) in the counter-stereotypic condition (*p* = .733, *d* = .06), suggesting some attenuation of boys’ gender-typed biases around play style and playmates if they are already aware that the child possesses a counter-stereotypic play style. However, pairwise comparisons examining differences in girls’ and boys’ gender-based social exclusion scores across conditions were not significant (*p*s > .05).

## Discussion

The present study investigated the impact of stereotypic and counter-stereotypic children presented in children’s magazines on participants’ gender flexibility around gender-typed toy preferences for themselves and others, playmate choices, and endorsement of gender-based social exclusion. Most of the hypotheses were fully supported and a number of important patterns were observed. Participants did *not* demonstrate more gender flexible attitudes in their *own* preferences for gender-typed toys (i.e., girls preferred feminine toys and boys preferred masculine toys) after exposure to counter-stereotypic content. However, participants in the counter-stereotypic condition did demonstrate more gender flexible attitudes toward the toy play of *other* boys and girls, labeling masculine toys and feminine toys as appropriate for both boys and girls more often than participants in the stereotypic condition.

In addition, we observed a strong preference for same-gender playmates over other-gender playmates among participants in the stereotypic condition, but we observed no preference for same-gender playmates over other-gender playmates among participants in the counter-stereotypic condition. This choice of playmate in the counter-stereotypic condition appeared to be driven more by the type of toy play being modeled by the child than by the child’s gender. Using a more explicit indicator of social exclusion, we found that in the stereotypic condition, boys were more supportive of gender-based exclusion than were girls. Meanwhile in the counter-stereotypic condition, there were no significant differences between boys and girls in their endorsement of gender-based exclusion.

Our study represents the first known investigation of the impact of counter-stereotypic peers pictured in children’s magazines on children’s gender flexibility around toy and playmate attitudes and preferences. On the whole, our findings suggest that exposure to counter-stereotypic content that challenges gender-typed toy play may be a useful strategy for attenuating gender-typed attitudes and behavior in young children, at least encouraging more flexible thinking around the gender-typed toy play of other boys and girls.

In contrast to Green et al. (2004) who used fictional characters to display counter-stereotypic gender models, we found that gender counter-stereotypic peers shifted boys’ as well as girls’ gender-typed attitudes. This suggests that pictured examples of actual children engaged in counter-stereotypic toy play (in a media format) may be more effective at changing children’s gender-typed attitudes than the use of fictional characters. The use of actual children may also facilitate greater perceived behavioral similarity with the peers, which has been linked to the potential countering of gender-typed attitudes and behaviors (Martin et al. 2011). Children may have perceived themselves as similar to the other-gender peer in the counter-stereotypic condition if the peer displayed similar toy preferences to themselves, and this possibility should be explored further in future research.

Although we hypothesized that exposure to the counter-stereotypic peers in the magazine would amplify children’s gender flexibility, it is perhaps unsurprising that children’s *own* preferences for toys remained gender-typed. This pattern is consistent with previous research, which has shown children’s gender attitudes are easier to manipulate than their behaviors (for example, Bigler and Liben 1990, in the context of gender-typed occupations). Children’s own gender-related attitudes may be less flexible because of the increased risk of peer rejection associated with preferences (and behaviors) that break gender norms. Therefore, more intensive interventions with peer reinforcement may be required to effectively change children’s own gender-typed toy preferences. This underscores the idea that a single exposure to gender atypical toy play would not affect deeply entrenched attitudes (Weisgram et al. 2014; Wong and Hines 2015), a point to which we return in the Limitations section.

The fact that such exposure did alter attitudes around *other’s* toy play was consistent with expectations and warrants further consideration. There is some evidence to suggest that exposure to non-traditional toy play in television commercials can increase gender flexible attitudes around toy play in children between 6 and 8 years-old (Pike and Jennings 2005). Given the role of toy play style in directing children’s social and cognitive development (Alexander 2003; Alexander and Hines 2002; Blakemore et al. 2009; Blakemore and Centers 2005; De Lisi and Wolford 2002), it behooves scholars and practitioners to understand how we can harness toy play to maximize potential and growth for all children. Furthermore, engagement with a wider variety of toys that cross traditional gender lines may increase the possibility for more cross-gender friendships to develop and be sustained, which has also shown to be beneficial for children’s development (Fabes et al. 2003).

### Limitations and Future Research Directions

Although we reported some interesting results regarding the gender flexibility of young children, our study is not without its limitations. First, we recognize the impact of the present study may be limited by the fact that we did not include a control condition against which to compare the direction of the observed effects. In future research, we would recommend a comparison against a peer playing with a gender-neutral toy (e.g., a puzzle) as well as against a non-exposure condition, which would reflect a truer baseline for gender flexible attitudes and behaviors. Furthermore, future research should standardize the images of the peers across conditions. Future research should also directly compare exposure to life-like peers with storybook characters to examine whether these images affect gender flexibility to different extents.

Second, we examined behavioral intentions in the context of hypothetical scenarios and contrived stimuli presented to children. An important next step in this program of research would be to examine toy and playmate preferences in the context of viewing gender counter-stereotypic peers in magazines in more natural settings where actual behavior can be observed.

Third, we presented participants with one exposure to a single magazine page and the impact on gender attitudes and preferences was measured immediately afterwards. This design was adopted with the intention of providing a snapshot of how media can impact children’s gender attitudes and preferences. Clearly a more intensive and regular intervention using counter-stereotypic peers would be necessary for long-lasting change, and future research is required in order to examine the long-term impact of such an intervention program. Such research would also determine whether the effects observed are due to priming or to more substantial changes to children’s understanding of and adherence to gender stereotypes.

Fourth, our relatively small sample size limited our ability to detect small and moderate effects of the magazine exposure, and it also precluded us from making age-based comparisons. Analyses revealed that gender flexibility as measured by gender-typed toy play, own gender-typed toy preference, and gender-based exclusion (boys only) was correlated with age. However, between the ages of 4–7 years, children undergo considerable changes in their understanding of and adherence to gender stereotypes. Rigidity and adherence to stereotypes appears to increase linearly from 3 to 6 years-old and begins to decline thereafter when gender flexibility emerges (Serbin and Sprafkin 1986; Signorella et al. 1993; Trautner et al. 2005). Future research should include a larger sample size to allow a thorough examination of developmental changes in behavioral (e.g. social exclusion, playmate preference) and attitudinal aspects of gender flexibility, as well as how these change in response to the peers across the age group studied here (i.e., compare the impact of the peers among children aged 4-, 5-, 6- and 7-years-old). Future research could also include measures of understanding of gender, such as gender constancy, to capture the differential impact of the peers depending on the child’s stage of gender development.

Moreover, future research should also examine the differential effect of exposure to gender stereotypic and counter-stereotypic children pictured in media across this age range. For example, research on encoding and memory processes has found that young children misremember or incorrectly process gender counter-stereotypic information to match their pre-existing gender schema (Liben and Signorella 1980; Martin and Halverson 1983; Signorella and Liben 1984). This research would suggest that a single exposure to gender counter-stereotypic children pictured in magazines or other media would have a stronger impact among the older children in our sample. This possibility warrants further study.

We also limited the playmate choice and social exclusion measures to ask about the children presented to the participants in the magazine. This was done in order to enhance the realistic nature of the scenarios, but it did mean the specific children targeted in the social exclusion scenarios varied by condition. Future research could adapt the methods employed here to include a variety of social exclusion scenarios, with new targets in the social exclusion scenario, in addition to those viewed in the magazines, in order to improve experimental control and test the generalizability of this finding to new children and social situations. It would also be beneficial to include an additional response option of “both” in the playmate choice measure to allow children to express a preference for playing with both girls and boys, instead of restricting their response to choosing one gender over the other, which may be masking children’s gender flexible preferences. The PPPSI (Pasterski et al. 2011) could also be included in future research to gain more detailed information about children’s play style and playmate preferences beyond what the present study was able to obtain.

It is noteworthy that the effect of counter-stereotypic peers on participant’s attitudes toward gender-typed toy play and playmate preference was the same across boys and girls. In fact, for endorsement of gender-based exclusion, counter-stereotypic peers brought boys’ and girls’ attitudes more in line with one another. This attenuation of gender bias in younger boys is, therefore, especially revealing. It could be argued that it is easier to change girls’ gender-related attitudes and behaviors, compared to boys’, because greater resistance to gender-atypical attitudes and behavior is more often observed among boys (Bussey and Perry 1982; Mulvey and Killen 2015). Furthermore, masculine traits, toys, and behaviors are generally accepted as being of higher status than their feminine counterparts, and research has shown that children are aware of these status differences (Ruble et al. 2006. For that reason, it may be easier to persuade girls to move toward masculine toy preferences, for instance, than persuading boys to choose feminine toys (Blakemore 2003, Horn 2008). Because boys and girls responded to the counter-stereotypic peers similarly in the present study, the use of pictures of actual children in a magazine format may overcome some of the difficulties in adjusting boys’ gender-typed attitudes. However, it would be interesting for future studies to examine the social status associated with masculine and feminine toys and activities in tempering the impact of stereotypic and counter-stereotypic peers on children’s gender flexibility.

### Practice Implications

The findings of the present study have several implications. First, these findings suggest it is possible to shift children toward more gender flexible attitudes and change children’s views on gender-related play. This possibility counters lay beliefs that gender segregation and gendered toy preference is inevitable in young children, and it adds to literature emphasizing the potential for change in children’s attitudes about gender-related play and friendships.

In particular, our findings suggest more regular exposure to counter-stereotypic content in the media could be an effective strategy to promote gender flexibility and combat gender-related bullying (Bigler 1999; Bussey and Perry 1982). That such an acute exposure shifted attitudes, underscores the impact that repeated exposure to gender stereotypical media can have on young children. These findings suggest that presenting children with images of counter-stereotypic peers through magazines could be used to encourage children to play with their own and other-gender toys, play in mixed-gender groups, and reduce gender-based social exclusion and bullying for both gender-typical and gender-atypical children.

Educators, parents, and policymakers might benefit from the present research and the approach tested to increase gender flexibility in children. This exposure technique could be extended for use in the classroom by providing more regular exposure to counter-stereotypic peers in children’s media through a series of magazine articles, or news stories, that feature such children. Children could also be asked to model and create their own resources. Moreover, our research shows that children consider both play style and gender when selecting a playmate. This finding suggests that highlighting behavioral similarities in children could encourage mixed-gender play. We suggest encouraging mixed-gender play by teachers and parents, despite the apparent gender segregation during play, because boys and girls are willing to play with one another if they possess similar toy and play style interests.

### Conclusion

Exposure to gender counter-stereotypic peers in a magazine format increases gender flexibility among young children. Specifically, children exposed to counter-stereotypic peers were more flexible in their attitudes toward what other children could play with and were more likely to choose an other-gender playmate, using play style as a guide more so than the playmate’s gender. Moreover, boys’ stronger endorsement of social exclusion in the stereotypic condition was attenuated in the counter-stereotypic condition. The results of the present study not only underscore the impact of media (specifically print media) on children’s early understanding of gender and conformity to gender stereotypes, but also highlight the potential use of media to challenge and disrupt gender-typed toy choices and playgroups in young children. In particular, this research highlights the potential use of counter-stereotypic same-age peers in children’s print media to normalize counter-stereotypic attitudes, and perhaps behaviors, as an important avenue for future research and intervention. On the whole, these results suggest that the observed play style and toy preferences of others could be used as a gateway to gender desegregation in children. We hope the present study will inspire further investigations of this possibility in children.
